# Social cognition impairment in genetic frontotemporal dementia within the GENFI cohort

**DOI:** 10.1016/j.cortex.2020.08.023

**Published:** 2020-12

**Authors:** Lucy L. Russell, Caroline V. Greaves, Martina Bocchetta, Jennifer Nicholas, Rhian S. Convery, Katrina Moore, David M. Cash, John van Swieten, Lize Jiskoot, Fermin Moreno, Raquel Sanchez-Valle, Barbara Borroni, Robert Laforce, Mario Masellis, Maria Carmela Tartaglia, Caroline Graff, Emanuela Rotondo, Daniela Galimberti, James B. Rowe, Elizabeth Finger, Matthis Synofzik, Rik Vandenberghe, Alexandre de Mendonça, Fabrizio Tagliavini, Isabel Santana, Simon Ducharme, Chris Butler, Alex Gerhard, Johannes Levin, Adrian Danek, Markus Otto, Jason D. Warren, Jonathan D. Rohrer, the Genetic FTD Initiative, GENFI, Martin N. Rossor, Nick C. Fox, Ione O.C. Woollacott, Rachelle Shafei, Carolin Heller, Rita Guerreiro, Jose Bras, David L. Thomas, Simon Mead, Lieke Meeter, Jessica Panman, Janne Papma, Jackie Poos, Rick van Minkelen, Yolanda Pijnenburg, Myriam Barandiaran, Begoña Indakoetxea, Alazne Gabilondo, Mikel Tainta, Maria de Arriba, Ana Gorostidi, Miren Zulaica, Jorge Villanua, Zigor Diaz, Sergi Borrego-Ecija, Jaume Olives, Albert Lladó, Mircea Balasa, Anna Antonell, Nuria Bargallo, Enrico Premi, Maura Cosseddu MPsych, Stefano Gazzina, Alessandro Padovani, Roberto Gasparotti, Silvana Archetti, Sandra Black, Sara Mitchell, Ekaterina Rogaeva, Morris Freedman, Ron Keren, Daid Tang-Wai, Linn Öijerstedt, Christin Andersson, Vesna Jelic, Hakan Thonberg, Andrea Arighi, Chiara Fenoglio, Elio Scarpini, Giorgio Fumagalli, Thomas Cope, Carolyn Timberlake, Timothy Rittman, Christen Shoesmith, Robart Bartha, Rosa Rademakers, Carlo Wilke, Hans-Otto Karnarth, Benjamin Bender, Rose Bruffaerts, Philip Vandamme, Mathieu Vandenbulcke, Catarina B. Ferreira, Gabriel Miltenberger, Carolina Maruta MPsych, Ana Verdelho, Sónia Afonso, Ricardo Taipa, Paola Caroppo, Giuseppe Di Fede, Giorgio Giaccone, Cristina Muscio, Sara Prioni, Veronica Redaelli, Giacomina Rossi, Pietro Tiraboschi, Diana Duro NPsych, Maria R. Almeida, Miguel Castelo-Branco, Maria J. Leitão, Miguel Tabuas-Pereira, Beatriz Santiago, Serge Gauthier, Pedro Rosa-Neto, Michele Veldsman, Paul Thompson, Tobias Langheinrich, Catharina Prix, Tobias Hoegen, Elisabeth Wlasich, Sandra Loosli, Sonja Schonecker, Elisa Semler, Sarah Anderl-Straub

**Affiliations:** ab Dementia Research Centre, Department of Neurodegenerative Disease, UCL Institute of Neurology, Queen Square, London, UK; ac Dementia Research Institute, Department of Neurodegenerative Disease, UCL Institute of Neurology, Queen Square, London, UK; ad Neuroimaging Analysis Centre, Department of Brain Repair and Rehabilitation, UCL Institute of Neurology, Queen Square, London, UK; ae MRC Prion Unit, Department of Neurodegenerative Disease, UCL Institute of Neurology, Queen Square, London, UK; af Department of Neurology, Erasmus Medical Centre, Rotterdam, Netherlands; ag Department of Clinical Genetics, Erasmus Medical Centre, Rotterdam, Netherlands; ah Amsterdam University Medical Centre, Amsterdam VUmc, Amsterdam, Netherlands; ai Cognitive Disorders Unit, Department of Neurology, Donostia University Hospital, San Sebastian, Gipuzkoa, Spain; aj Neuroscience Area, Biodonostia Health Research Insitute, San Sebastian, Gipuzkoa, Spain; ak OSATEK, University of Donostia, San Sebastian, Gipuzkoa, Spain; al CITA Alzheimer, San Sebastian, Gipuzkoa, Spain; am Alzheimer's Disease and Other Cognitive Disorders Unit, Neurology Service, Hospital Clínic, Barcelona, Spain; an Imaging Diagnostic Center, Hospital Clínic, Barcelona, Spain; ao Centre for Neurodegenerative Disorders, Neurology Unit, Department of Clinical and Experimental Sciences, University of Brescia, Brescia, Italy; ap Neuroradiology Unit, University of Brescia, Brescia, Italy; aq Biotechnology Laboratory, Department of Diagnostics, Spedali Civili Hospital, Brescia, Italy; ar Sunnybrook Health Sciences Centre, Sunnybrook Research Institute, University of Toronto, Toronto, Canada; as Tanz Centre for Research in Neurodegenerative Diseases, University of Toronto, Toronto, Canada; at Baycrest Health Sciences, Rotman Research Institute, University of Toronto, Toronto, Canada; au The University Health Network, Toronto Rehabilitation Institute, Toronto, Canada; av The University Health Network, Krembil Research Institute, Toronto, Canada; aw Department of Geriatric Medicine, Karolinska University Hospital-Huddinge, Stockholm, Sweden; ax Department of Clinical Neuroscience, Karolinska Institutet, Stockholm, Sweden; ay Division of Clinical Geriatrics, Karolinska Institutet, Stockholm, Sweden; az Center for Alzheimer Research, Divison of Neurogeriatrics, Karolinska Institutet, Stockholm, Sweden; ba Fondazione IRCCS Ca’ Granda Ospedale Maggiore Policlinico, Neurodegenerative Diseases Unit, Milan, Italy; bb University of Milan, Centro Dino Ferrari, Milan, Italy; bc Department of Neurosciences, Psychology, Drug Research and Child Health (NEUROFARBA), University of Florence, Florence, Italy; bd Department of Clinical Neurosciences, University of Cambridge, Cambridge, UK; be Department of Clinical Neurological Sciences, University of Western Ontario, London, Ontario Canada; bf Department of Medical Biophysics, The University of Western Ontario, London, Ontario, Canada; bg Centre for Functional and Metabolic Mapping, Robarts Research Institute, The University of Western Ontario, London, Ontario, Canada; bh Department of Neuroscience, Mayo Clinic, Jacksonville, FL, USA; bi Department of Neurodegenerative Diseases, Hertie-Institute for Clinical Brain Research and Center of Neurology, University of Tübingen, Tübingen, Germany; bj Center for Neurodegenerative Diseases (DZNE), Tübingen, Germany; bk Division of Neuropsychology, Hertie-Institute for Clinical Brain Research and Center of Neurology, University of Tübingen, Tübingen, Germany; bl Department of Diagnostic and Interventional Neuroradiology, University of Tübingen, Tübingen, Germany; bm Laboratory for Cognitive Neurology, Department of Neurosciences, KU Leuven, Leuven, Belgium; bn Neurology Service, University Hospitals Leuven, Belgium, Laboratory for Neurobiology, VIB-KU Leuven Centre for Brain Research, Leuven, Belgium; bo Geriatric Psychiatry Service, University Hospitals Leuven, Belgium; bp Neuropsychiatry, Department of Neurosciences, KU Leuven, Leuven, Belgium; bq Laboratory of Neurosciences, Institute of Molecular Medicine, Faculty of Medicine, University of Lisbon, Lisbon, Portugal; br Faculty of Medicine, University of Lisbon, Lisbon, Portugal; bs Laboratory of Language Research, Centro de Estudos Egas Moniz, Faculty of Medicine, University of Lisbon, Lisbon, Portugal; bt Department of Neurosciences and Mental Health, Centro Hospitalar Lisboa Norte - Hospital de Santa Maria & Faculty of Medicine, University of Lisbon, Lisbon, Portugal; bu Instituto Ciencias Nucleares Aplicadas a Saude, Universidade de Coimbra, Coimbra, Portugal; bv Neuropathology Unit and Department of Neurology, Centro Hospitalar do Porto - Hospital de Santo António, Oporto, Portugal; bw Fondazione IRCCS Istituto Neurologico Carlo Besta, Milano, Italy; bx Faculty of Medicine, University of Coimbra, Coimbra, Portugal; by Centre of Neurosciences and Cell Biology, Universidade de Coimbra, Coimbra, Portugal; bz Neurology Department, Centro Hospitalar e Universitario de Coimbra, Coimbra, Portugal; ca Alzheimer Disease Research Unit, McGill Centre for Studies in Aging, Department of Neurology & Neurosurgery, McGill University, Montreal, Québec, Canada; cb Translational Neuroimaging Laboratory, McGill Centre for Studies in Aging, McGill University, Montreal, Québec, Canada; cc Nuffield Department of Clinical Neurosciences, Medical Sciences Division, University of Oxford, Oxford, UK; cd Faculty of Biology, Medicine and Health, Division of Neuroscience and Experimental Psychology, University of Manchester, Manchester, UK; ce Neurologische Klinik, Ludwig-Maximilians-Universität München, Munich, Germany; cf Department of Neurology, University of Ulm, Ulm, Germany; a Dementia Research Centre, Department of Neurodegenerative Disease, London, UK; b Department of Medical Statistics, London School of Hygiene and Tropical Medicine, London, UK; c Institute of Prion Disease, UCL Queen Square Institute of Neurology, London, UK; d Centre for Medical Image Computing, Department of Medical Physics and Biomedical Engineering, University College London, London, UK; e Department of Neurology, Erasmus Medical Centre, Rotterdam, Netherlands; f Cognitive Disorders Unit, Department of Neurology, Donostia University Hospital, San Sebastian, Gipuzkoa, Spain; g Alzheimer's Disease and Other Cognitive Disorders Unit, Neurology Service, Hospital Clínic, Barcelona, Spain; h Centre for Neurodegenerative Disorders, Neurology Unit, Department of Clinical and Experimental Sciences, University of Brescia, Brescia, Italy; i Clinique Interdisciplinaire de Mémoire, Département des Sciences Neurologiques du CHU de Québec, Université Laval, Québec, Canada; j Sunnybrook Health Sciences Centre, Sunnybrook Research Institute, University of Toronto, Toronto, Canada; k Tanz Centre for Research in Neurodegenerative Diseases, University of Toronto, Toronto, Canada; l Department of Geriatric Medicine, Karolinska University Hospital-Huddinge, Stockholm, Sweden; m University of Milan, Centro Dino Ferrari, Milan, Italy; n Fondazione Ca’ Granda, IRCCS Ospedale Policlinico, Milan, Italy; o Department of Clinical Neurosciences, University of Cambridge, Cambridge, UK; p Department of Clinical Neurological Sciences, University of Western Ontario, London, Ontario, Canada; q Department of Neurodegenerative Diseases, Hertie-Institute for Clinical Brain Research and Center of Neurology, University of Tübingen, Tübingen, Germany; r Laboratory for Cognitive Neurology, Department of Neurosciences, KU Leuven, Leuven, Belgium; s Faculty of Medicine, University of Lisbon, Lisbon, Portugal; t Fondazione Istituto di Ricovero e Cura a Carattere Scientifico Istituto Neurologica Carlo Besta, Milano, Italy; u Faculty of Medicine, University of Coimbra, Coimbra, Portugal; v Department of Psychiatry, McGill University, Montreal, Québec, Canada; w Department of Clinical Neurology, University of Oxford, Oxford, UK; x Division of Neuroscience and Experimental Psychology, Wolfson Molecular Imaging Centre, University of Manchester, Manchester, UK; y Departments of Geriatric Medicine and Nuclear Medicine, University of Duisburg- Essen, Germany; z Department of Neurology, Ludwig-Maximilians-University, Munich, Germany; aa Department of Neurology, University of Ulm, Ulm, Germany

**Keywords:** Frontotemporal dementia, Theory of mind, Emotion processing, Faux pas, Facial emotion recognition, C9orf72, Progranulin, MAPT

## Abstract

A key symptom of frontotemporal dementia (FTD) is difficulty interacting socially with others. Social cognition problems in FTD include impaired emotion processing and theory of mind difficulties, and whilst these have been studied extensively in sporadic FTD, few studies have investigated them in familial FTD. Facial Emotion Recognition (FER) and Faux Pas (FP) recognition tests were used to study social cognition within the Genetic Frontotemporal Dementia Initiative (GENFI), a large familial FTD cohort of *C9orf72, GRN,* and *MAPT* mutation carriers. 627 participants undertook at least one of the tasks, and were separated into mutation-negative healthy controls, presymptomatic mutation carriers (split into early and late groups) and symptomatic mutation carriers. Groups were compared using a linear regression model with bootstrapping, adjusting for age, sex, education, and for the FP recognition test, language. Neural correlates of social cognition deficits were explored using a voxel-based morphometry (VBM) study. All three of the symptomatic genetic groups were impaired on both tasks with no significant difference between them. However, prior to onset, only the late presymptomatic *C9orf72* mutation carriers on the FER test were impaired compared to the control group, with a subanalysis showing differences particularly in fear and sadness. The VBM analysis revealed that impaired social cognition was mainly associated with a left hemisphere predominant network of regions involving particularly the striatum, orbitofrontal cortex and insula, and to a lesser extent the inferomedial temporal lobe and other areas of the frontal lobe. In conclusion, theory of mind and emotion processing abilities are impaired in familial FTD, with early changes occurring prior to symptom onset in *C9orf72* presymptomatic mutation carriers. Future work should investigate how performance changes over time, in order to gain a clearer insight into social cognitive impairment over the course of the disease.

## 1 Introduction

The impairment of social skills is one of the most prominent symptoms experienced by people with frontotemporal dementia (FTD) (Adenzato, Cavallo, & Enrici, 2010; Kumfor and Piguet, 2012). The different neural processes that underlie such skills are generally grouped together within the term ‘social cognition’ (Adolphs, 2009), and include a number of abilities that have been shown to be impaired in FTD, including recognition of others' emotions, and ‘theory of mind’, the ability to understand that others have thoughts and beliefs (Gregory et al., 2002; Lough and Hodges, 2002; Rosen et al., 2006; Adenzato et al., 2010; Omar, Rohrer, Hailstone, & Warren, 2011; Kumfor and Piguet, 2012).

Whilst there have been a number of studies exploring these skills in sporadic FTD, few have focused on people with the genetic forms of FTD, characterized usually by mutations in the progranulin (*GRN*), tau (*MAPT*) and chromosome 9 open reading frame 72 (*C9orf72*) genes (Jiskoot et al., 2016, 2018, Cheran et al., 2019). So far, these studies have been relatively small and often focused on one (Cheran et al., 2019) or two (Jiskoot et al., 2016, 2018) of the genetic groups, showing change only in specific questionnaires, or when groups were followed longitudinally.

The Genetic FTD Initiative (GENFI) is an international genetic FTD cohort study, aimed at investigating early biomarkers, including measures of cognition (Rohrer et al., 2015). Using this cohort we therefore aimed to assess emotion processing and theory of mind abilities in a large cohort of presymptomatic and symptomatic individuals with mutations in the *C9orf72*, *GRN* and *MAPT* genes, with the hypothesis that social cognitive deficits would become apparent only late in the presymptomatic period or when symptomatic.

## 2 Methods

We report how we determined our sample size, all data exclusions (if any), all inclusion/exclusion criteria, whether inclusion/exclusion criteria were established prior to data analysis, all manipulations, and all measures in the study.

### 2.1 Participants

Participants were recruited from the fourth data freeze of the GENFI study including sites in the UK, Canada, Sweden, Netherlands, Belgium, Spain, Portugal, Italy and Germany. Of the 680 participants consecutively enrolled in the study, 627 undertook at least one test of social cognition: 246 who tested negative for the mutation within the family, and therefore acted as the controls, 159 *C9orf72* expansion carriers, 155 GRN mutation carriers, and 67 *MAPT* mutation carriers (Table 1). Mutation carriers were classified as either symptomatic or presymptomatic based on clinician judgement. Participants were only classified as symptomatic if the clinician judged that symptoms were present, consistent with a diagnosis of a degenerative disorder, and progressive in nature (Table S1).

**Table 1:** Demographics and scores for the Facial Emotion Recognition (FER) and Faux Pas (FP) recognition tests. N is the number of participants. Mean (standard deviation) shown for age, education and cognitive test scores. *As a slightly different number of participants attempted each test in some of the subgroups, the mean (standard deviation) sex, age, education, MMSE and FTLD-CDR varied between those that did the FER test and those that did the FP recognition test – these are shown underneath in italics for the FP recognition test if different.*

		N (FER)/(FP)	Sex (% male)	Age (years)	Education (years)	MMSE (/30)	FTLD-CDR (Sum of boxes)	FER test score (/35)	FER subscores by emotion (each score out of 5)							FP recognition test score (/40)
									Neutral	Happy	Surprise	Disgust	Fear	Anger	Sadness	
Healthy controls		246/245	42	46.0 (12.8)	14.3 (3.5)	29.4 (1.2)	.2 (.6)	28.5 (3.3)	4.8 (.5)	5.0 (.2)	4.5 (.9)	4.0 (1.0)	3.0 (1.4)	3.9 (.9)	3.5 (1.3)	35.1 (4.6)
*C9orf72*	Early presymptomatic	81/81	41	41.7 (10.1)	14.8 (2.5)	29.4 (1.0)	.3 (.6)	29.0 (2.9)	4.9 (.4)	5.0 (.0)	4.6 (.8)	3.8 (1.1)	3.1 (1.3)	3.9 (1.0)	3.8 (1.2)	35.0 (5.2)
	Late presymptomatic	25/24	36	56.3 (8.3) *56.5 (8.4)*	13.2 (3.9) *13.1 (3.9)*	28.7 (1.3) *28.7 (1.4)*	.4 (.9)	26.3 (3.5)	4.8 (.5)	5.0 (.0)	4.2 (1.2)	3.6 (1.2)	2.3 (1.2)	3.7 (1.1)	2.7 (1.4)	31.9 (7.5)
	Symptomatic	53/45	64 *62*	62.3 (8.0) *63.0 (8.0)*	13.0 (3.6) *13.0 (3.7)*	24.7 (4.9) *24.9 (5.2)*	9.3 (5.6) *9.2 (5.3)*	18.7 (6.9)	3.5 (1.8)	4.4 (1.2)	3.0 (1.6)	2.4 (1.5)	1.4 (1.3)	2.3 (1.5)	1.9 (1.5)	22.0 (9.9)
*GRN*	Early presymptomatic	93/93	35	41.3 (9.1)	15.0 (3.7)	29.5 (.8)	.1 (.2)	29.3 (3.2)	4.9 (.4)	5.0 (.0)	4.6 (.9)	4.0 (1.0)	3.2 (1.3)	3.9 (1.0)	3.8 (1.2)	36.3 (4.3)
	Late presymptomatic	29/30	48	60.5 (6.6) *60.3 (6.5)*	14.4 (3.2) *14.3 (3.1)*	29.2 (1.1)	.2 (.6)	28.4 (4.2)	4.8 (.7)	5.0 (.2)	4.5 (.7)	3.8 (1.2)	2.9 (1.3)	3.9 (1.2)	3.6 (1.1)	35.6 (3.7)
	Symptomatic	32/22	53 *41*	64.2 (8.4) *62.9 (7.9)*	11.6 (3.6) *11.4 (3.2)*	21.8 (6.3) *21.8 (7.1)*	8.6 (5.5) *8.6 (5.6)*	20.0 (7.2)	3.2 (1.8)	4.4 (.9)	3.1 (1.4)	3.0 (1.7)	2.0 (1.7)	2.9 (1.4)	1.9 (1.6)	18.7 (12.2)
*MAPT*	Early presymptomatic	37/37	35	36.1 (8.0)	14.8 (2.7)	29.7 (.8)	.3 (.6)	29.5 (3.0)	4.8 (.4)	5.0 (.0)	4.5 (.9)	4.1 (.9)	3.5 (1.6)	3.9 (1.0)	3.6 (1.3)	35.2 (4.5)
	Late presymptomatic	12/12	42	51.2 (10.2)	14.0 (3.4)	29.3 (1.0)	.2 (.6)	29.4 (2.2)	4.8 (.4)	5.0 (.0)	4.8 (.5)	4.0 (1.2)	3.2 (1.2)	4.2 (.7)	3.5 (.8)	34.7 (4.5)
	Symptomatic	18/12	56 *50*	59.8 (6.0) *59.7 (5.7)*	14.6 (3.6) *15.1 (4.0)*	23.2 (6.5) *25.8 (3.3)*	9.0 (5.3) *8.5 (5.5)*	22.3 (6.6)	4.3 (1.6)	4.8 (.5)	3.3 (1.6)	2.6 (1.7)	2.1 (1.5)	2.6 (1.6)	2.7 (1.1)	29.2 (7.0)

The presymptomatic carriers were further split into those further than five years from estimated symptom onset (based on the mean age at onset in the family), called the ‘early’ group, and those within five years of estimated onset, called the ‘late’ group. Diagnoses in the symptomatic group were as follows: *MAPT* mutation carriers, 17 bvFTD, 1 other; *GRN* mutation carriers, 15 bvFTD, 16 primary progressive aphasia (PPA), 1 other; *C9orf72* expansion carriers, 38 bvFTD, 10 FTD with amyotrophic lateral sclerosis, 1 PPA, 1 progressive supranuclear palsy and 3 other.

All participants underwent the standardized GENFI clinical assessment including medical history, physical examination, the Mini-Mental State Examination (MMSE), and the Clinical Dementia Rating Scale with the National Alzheimer Coordinating Centre FTLD sum of boxes score (FTLD-CDR-SOB). Demographics are shown in Table 1. There was a significant difference in sex between the groups (*p* = .018): the symptomatic *C9orf72* mutation carriers had a significantly higher percentage of men than the early and late *C9orf72* mutation carriers and the control group (*p* = .013, *p* = .002 and *p* = .001 respectively). There was also a significant difference in age between the groups: all early presymptomatic mutation carriers were significantly younger than the control group (all *p* < .001), and all late presymptomatic mutation carriers and symptomatic mutation carriers were significantly older than controls (all *p* < .001) except for the late *MAPT* mutation carriers in which no difference was observed (*p* = .239). There were also differences between the groups in education: the symptomatic *C9orf72* and *GRN* mutation carriers had significantly lower levels of education than the control group did (*p* = .007 and *p* < .001 respectively). No significant differences in disease severity were observed between the symptomatic genetic groups or between the late presymptomatic groups, based on their FTLD-CDR-SOB. However, the early GRN presymptomatic mutation carrier did have a significantly lower FTLD-CDR-SOB scores than the other two early groups.

### 2.2 Testing of social cognition

Social cognition was tested in the GENFI cohort using the shortened version of the Social Cognition and Emotional Assessment, known as the mini-SEA (Bertoux et al., 2012; Funkiewiez, Bertoux, de Souza, Lévy, & Dubois, 2012) which consists of a test of facial emotion recognition and a test of theory of mind. It was designed specifically for people with FTD, with initial studies showing deficits in FTD compared with healthy controls, with people with Alzheimer's disease, and also those with major depressive disorder (Guevara et al., 2015; Narme, Mouras, Roussel, Devendeville, & Godefroy, 2013; Torralva, Gleichgerrcht, Torres Ardila, Roca, & Manes, 2015).

#### 2.2.1 Experiment 1: facial emotion recognition (FER) test

The FER test is a shortened version of the standard Ekman faces task (Ekman, Ellsworth, Friesen, Goldstein, & Krasner, 1972), with participants asked to recognise whether faces are showing one of either six universal emotions (happiness, surprise, anger, fear, disgust and sadness) or a neutral expression. Participants are presented with 35 different faces (five items for each emotion) and are required to select the correct emotional label that matches the emotion of the face.

#### 2.2.2 Experiment 2: faux pas (FP) recognition test

The FP recognition test contains a series of 10 short cartoon stories describing scenarios involving social inconveniences, known as ‘faux pas’; five of the stories contain a faux pas, the other five do not. The task requires individuals to be able to infer another's thoughts or beliefs. A structured questionnaire asks how and why the social faux pas has occurred. Participants can score a maximum of 40 on this task, 10 points for the control stories and 30 points for the faux pas stories.

### 2.3 Statistical analysis

In the control group, we explored the relationship of the FER and FP recognition tests to age (Spearman rank correlation), sex (Mann–Whitney *U* test) and years of education (Spearman rank correlation). For the FP recognition test, we explored the effect of the different language versions using a linear regression.

Scores on the two social cognitive tests (and the individual emotion scores on the FER test) were compared between the groups using linear regression, adjusting for age, sex and education (and language for the FP recognition test) with 95% bias-corrected bootstrapped confidence intervals with 1000 repetitions (as the data was not normally distributed).

A subanalysis of the effect of phenotype was also performed using the same methodology as the main analysis: scores on the two social cognitive tests were compared between the different clinical syndromes within the symptomatic mutation carriers as well as with controls.

### 2.4 Imaging analysis

Participants underwent volumetric T1-weighted MRI using the GENFI protocol. A variety of 3T scanners were used across the sites: Siemens Trio, Siemens Skyra, Siemens Prisma, Phillips and General Electric. The scan protocols were designed at the start of the GENFI study to ensure that there was adequate matching between the scanners and the quality of the images. All scans were quality checked and those with movements or artefacts were removed. Furthermore, if any participants displayed moderate to severe vascular disease or any other brain lesions, they were also excluded from the analysis.

Voxel-based morphometry (VBM) was performed using Statistical Parametric Mapping (SPM) 12 software, version 6685 (www.fil.ion.ucl.ac.uk/spm), running under Matlab R2014a (Mathworks, USA). The T1-weighted images were normalized and segmented into grey matter (GM), white matter (WM) and cerebrospinal fluid (CSF) probability maps, by using standard procedures and the fast-diffeomorphic image registration algorithm (DARTEL) (Ashburner, 2007). GM segmentations were affine transformed into the Montreal Neurological Institute (MNI) space, modulated and smoothed using a Gaussian kernel with 6 mm full-width at half maximum before analysis. Finally, a mask was applied as reported in Ridgway et al., 2009. Study-specific templates were created based on the subjects included in the specific analysis. At each stage, all segmentations were reviewed visually. Total intracranial volume (TIV) was calculated using SPM (Malone et al., 2015).

In order to explore the relationship between performance on the tests and GM density, multiple regression models for each genetic group were used to correlate the GM tissue maps to the FER and FP performance in mutation carriers (both symptomatic and presymptomatic individuals combined). 319 scans were used for the FER analysis and 309 scans were used for the FP analysis (*C9orf72* expansion carriers: FER = 132, FP = 128, *GRN* mutation carriers: FER = 132, FP = 129, and *MAPT* mutation carriers: FER = 55, FP = 52) were included in the imaging analysis. Control participants were not included in any of the analysis. Age, sex, scanner type and TIV were included as nuisance covariates. The Family-Wise Error (FWE) rate for multiple comparisons correction was set at .05. If there were no findings at that strict level of correction, results were reviewed at an uncorrected *p* value of .001.

No part of the study procedure or analyses were pre-registered prior to the research being conducted. The conditions of our ethics approval do not permit public archiving of individual anonymised data. Readers seeking access to the data should contact the corresponding author. Access will be granted to named individuals in accordance with ethical procedures governing the reuse of sensitive data, including completion of a data sharing agreement. All stimuli and statistical code have been archived at: https://osf.io/m8yp7/?view_only=949ba796b549 4b7b87d37766adf840bf.

## 3 Results

### 3.1 Experiment 1: facial emotion recognition (FER) test

#### 3.1.1 Healthy controls

FER test score was not significantly correlated with either age (rho = −.12, *p* = .063) (Table S2) or education (rho = .13, *p* = .051) (Table S3) within the controls. However, there was a significant effect of sex (*p* = .031): mean (standard deviation) score overall in controls was 28.5 (3.3), with a higher score of 29.1 (3.1) in females (n = 143), compared with 28.2 (3.2) in males (n = 103).

Overall, controls scored between 19 and 34 out of a total possible score of 35, with cumulative frequency shown in Table S4. A cut-off score below the 5th percentile is commonly considered to be abnormal: for the FER test a score of below 23 would therefore be considered outside the normal range, with a score of 23 considered borderline abnormal.

#### 3.1.2 Mutation carriers

**Fig. 1:**
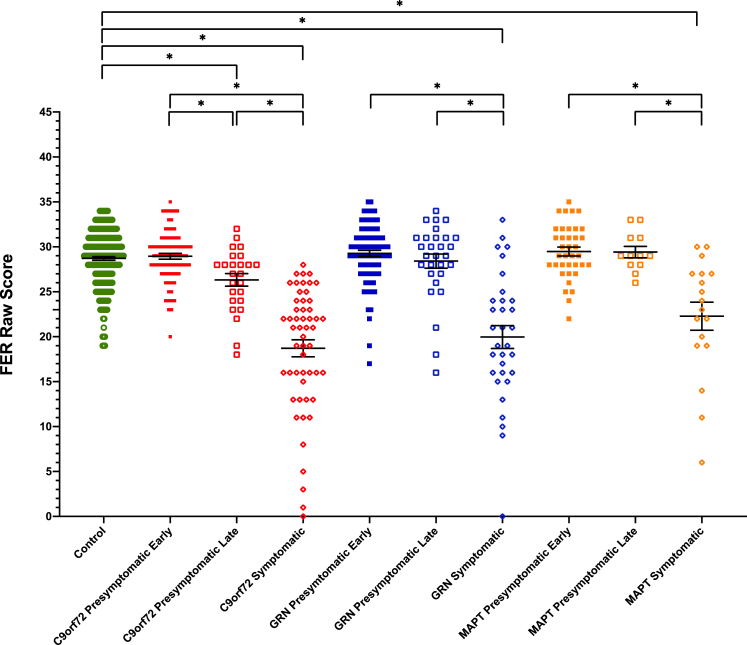
Facial Emotion Recognition test scores in each group. Significant differences from controls and within each genetic group are starred. Differences across genetic groups are not shown.

All of the three symptomatic mutation carrier groups scored significantly lower on the FER test compared with controls (Table 1, Table S4, Fig. 1): *C9orf72* mean 18.7 (standard deviation 6.9), *GRN* 20.0 (7.2) and *MAPT* 22.3 (6.6), with no significant difference between the disease groups.

Within each genetic group, scores were significantly lower in the symptomatic group compared with both the early and late presymptomatic groups (Table 1, Table S5, Fig. 1).

The *C9orf72* late presymptomatic group performed significantly lower than both the *C9orf72* early presymptomatic group and the controls (Table 1, Table S5, Fig. 1): late presymptomatic group 26.3 (3.5), early presymptomatic group 29.0 (2.9). No significant differences were seen between the other presymptomatic groups and controls.

#### 3.1.3 Phenotypic analysis

All phenotypic groups [bvFTD (19.6 {6.3}), PPA (22.0 {6.4}) and an FTD-ALS/ALS group (18.4 {8.1})] were significantly impaired on the FER test compared with controls, with no significant differences between any of the clinical syndromes (Table S6 and Table S7).

#### 3.1.4 Imaging analysis

**Fig. 2:**
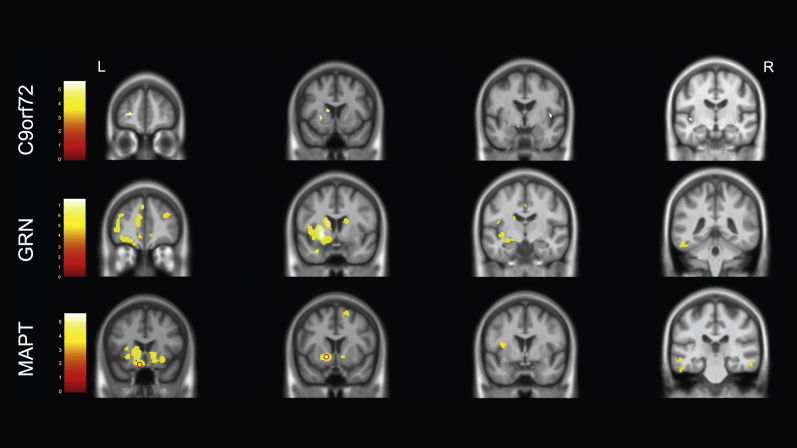
Neural correlates of performance on the Facial Emotion Recognition test. Results for C9orf72 and GRN groups are shown at *p* < .05, corrected for Family Wise Error whilst the results for the MAPT group are shown at *p* < .001 uncorrected (with the regions circled that are significant at *p* < .05 corrected for Family Wise Error). Results are shown on a study-specific T1-weighted MRI template in MNI space. Colour bars represent T-values.

In *C9orf72* mutation carriers, FER test score was positively associated with bilateral insula involvement, as well as atrophy in the left frontal lobe (middle frontal gyrus and orbitofrontal cortex), left basal ganglia (putamen and caudate) and right amygdala (Table S8, Fig. 2).

For the *GRN* mutation carriers, performance was positively correlated with a left hemisphere predominant network of areas involving the insula, frontal lobe, inferomedial temporal lobe, cingulate, basal ganglia (putamen and caudate) and thalamus (Table S8, Fig. 2).

In the *MAPT* mutation group FER test score positively correlated with two small clusters, one in the left basal ganglia and one in the left orbitofrontal cortex when correcting for multiple comparisons. At an uncorrected *p* value of <.001, there was also an association with the left insula and inferomedial temporal lobe as well as bilateral superior frontal and orbitofrontal regions (Table S8, Fig. 2).

#### 3.1.5 Subanalysis of performance on individual emotions

Identification of negative emotions (fear, anger, sadness and disgust) was in general worse than the recognition of positive ones (happiness and surprise) in each of the groups (including controls).

**Fig. 3:**
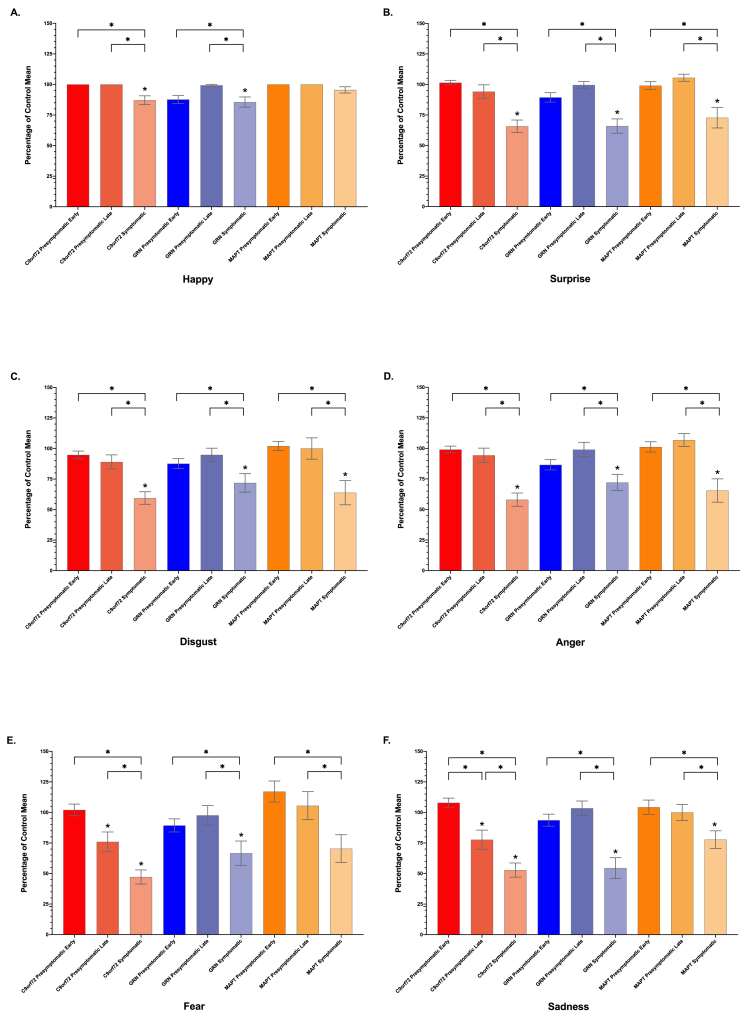
Facial Emotion Recognition test individual emotion subscores, shown as a percentage of the mean control score. Significant differences from controls are shown with a star at the top of the bar. Differences within each genetic group are shown with a bracket and star. Differences across genetic groups are not shown.

In almost all of the emotions, the symptomatic groups scored worse than controls (Table 1, Fig. 3). Only in the symptomatic *MAPT* mutation group for happiness and fear was there no significant difference.

In the presymptomatic groups, the *C9orf72* late presymptomatic group scored significantly lower than controls on both fear and sadness, but not on the other emotions (Fig. 3). No other significant differences were seen in the presymptomatic groups compared with controls.

### 3.2 Experiment 2: faux pas (FP) recognition test

#### 3.2.1 Healthy controls

As the FP recognition test was performed in eight different language versions, we initially compared the performance in controls across these language groups (Table S9). Significant differences were seen between the languages when adjusting for age, sex and education and therefore language was used as a covariate in the analysis.

FP recognition test score correlated with age (rho = −.21, *p* < .001) (Table S10) and education (rho = .18, *p* = .005) (Table S11) within the controls and there was an effect of sex (*p* = .006): mean (standard deviation) score overall in controls was 35.1 (4.6), with a higher score of 35.7 (4.7) in females (n = 142), compared with 34.3 (4.7) in males (n = 103).

Overall, controls scored between 19 and 40 out of a total possible score of 40, with cumulative frequency shown in Table S12. A cut-off score below the 5th percentile is commonly considered to be abnormal: for the FP recognition test a score of below 26 would therefore be considered outside the normal range, with a score of 26 considered borderline abnormal.

We also compared performance in controls (n = 245) across the FER and FP recognition tests, where there was a significant but weak correlation: rho = .20, *p* = .002.

#### 3.2.2 Mutation carriers

**Fig. 4:**
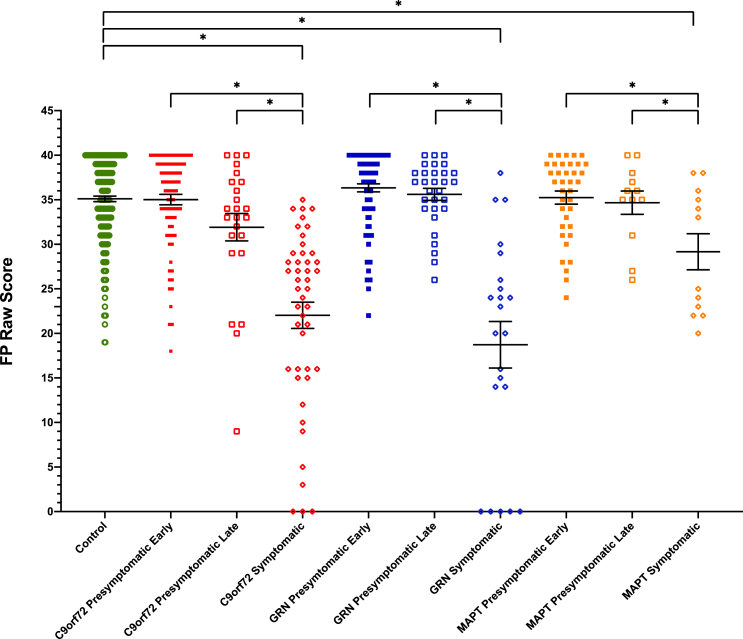
Faux Pas recognition test scores in each group. Significant differences from controls and within each genetic group are starred. Differences across genetic groups are not shown.

All of the three symptomatic mutation carrier groups scored significantly lower on the FP recognition test compared with controls (Table 1, Table S13, Fig. 4): *C9orf72* 22.0 (9.9), *GRN* 18.7 (12.2) and *MAPT* 29.2 (7.0), with significantly worse performance in the *C9orf72* and *GRN* groups compared with the *MAPT* group.

Within each genetic group, scores were significantly lower in the symptomatic group compared with both the early and late presymptomatic groups (Table 1, Table S13, Fig. 4).

No significant differences were seen between any of the presymptomatic groups and controls.

#### 3.2.3 Phenotypic analysis

All phenotypic groups [bvFTD (23.1 {10.0}), PPA (21.8 {14.6}) and an FTD-ALS/ALS group (21.1 {12.1})] were significantly impaired on the FP recognition test compared with controls, with no significant differences between any of the clinical syndromes (Table S14 and Table S15).

#### 3.2.4 Imaging analysis

**Fig. 5:**
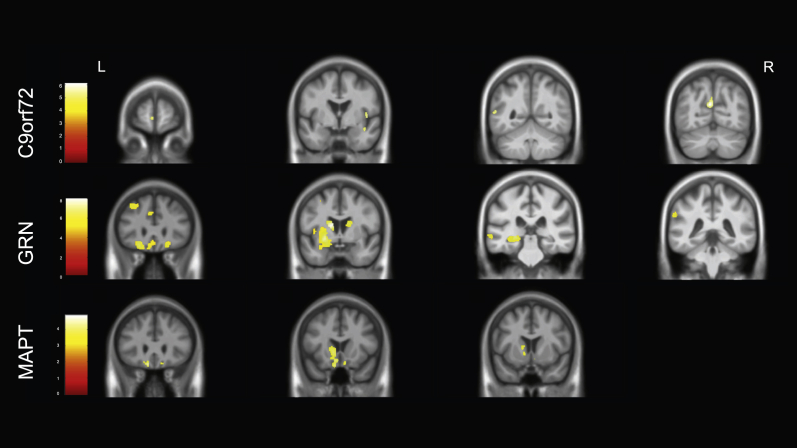
Neural correlates of performance on the Faux Pas recognition test. Results for *C9orf72* and *GRN* groups are shown at *p* < .05, corrected for Family Wise Error whilst the results for the *MAPT* group are shown at *p* < .001 uncorrected. Results are shown on a study-specific T1-weighted MRI template in MNI space. Colour bars represent T-values.

In the *C9orf72* mutation carriers, FP recognition test score was positively correlated with grey matter density in the left superior frontal gyrus, middle temporal gyrus, precuneus and lingual gyrus, as well as the insula and temporal lobe in the right hemisphere (Table S16, Fig. 5).

For the *GRN* mutation carriers, performance on the FP task was positively correlated with grey matter density in a predominantly left-sided network of regions including the basal ganglia, frontal lobe (orbitofrontal cortex, superior and inferior frontal gyri), insula, and temporal lobe (both medial i.e. amygdala and hippocampus, and other regions).

In the *MAPT* mutation carriers, there were no significant correlations when corrected for multiple comparisons. At an uncorrected *p*-value <.001, FP recognition test score was associated with atrophy in the left basal ganglia and left more than right orbitofrontal cortex mainly.

## 4 Discussion

In this study we have demonstrated that both the FER and FP recognition tests are able to detect social cognition deficits in familial forms of FTD during the symptomatic period, but only the FER test was able to detect presymptomatic deficits (particularly in the negative emotions of fear and sadness), specifically within *C9orf72* expansion carriers in proximity to symptom onset. Neural correlates varied across the different genetic groups with a left hemisphere predominant basal ganglia-orbitofrontal-insula network implicated across all three genetic groups on both tasks, except in the *C9orf72* group on the FP recognition test.

Investigation of mutation-negative members of families within the GENFI cohort has allowed us to study the performance of the mini-SEA in a larger healthy control population than previously, generating normative data across age, sex and education that can be used in other studies. We show a significant decline in performance with age with the theory of mind task consistent with the previous literature (Maylor, Moulson, Muncer, & Taylor, 2002; Pardini & Nichelli, 2009; Wang & Su, 2006). Prior studies have also shown an age-related decline in emotion processing (Mill, Allik, Realo, & Valk, 2009; Sullivan, Ruffman, & Hutton, 2007, pp. P53–P60; West et al., 2012), although in our study the correlation was weak with only a trend to significance (*p* = .063). A similar pattern was shown in the correlation with education (worse score with less years of education) with a weak but significant correlation on the FP recognition test and only a trend to significance in the FER test. Clearer differences were seen when comparing performance by sex, with females performing significantly better than males on both tasks as previously described (Hoffmann, Kessler, Eppel, Rukavina, & Traue, 2010; Kessels, Montagne, Hendriks, Perrett, & de Haan, 2014; Lee et al., 2002; Montagne, Kessels, Frigerio, de Haan, & Perrett, 2005). The results highlight the importance of adjusting for age, sex and education in analyses, particularly for theory of mind tasks.

Symptomatic mutation carriers in all groups performed significantly lower than their presymptomatic counterparts and the controls. This is in line with previous work in sporadic FTD demonstrating worse performance in FTD compared with controls using both the FER (Bertoux et al., 2014; Diehl-Schmid et al., 2007; Kumfor, Hazelton, Rushby, Hodges, & Piguet, 2019) and FP recognition tests (Bertoux, Funkiewiez, O'Callaghan, Dubois, & Hornberger, 2013; Funkiewiez et al., 2012).

Interestingly, there were no significant differences seen between phenotypes, with similar performance in the bvFTD, PPA and FTD-ALS/ALS groups on both the FER and FP recognition tests, and all three phenotypic groups being significantly worse than controls on both tasks. This is consistent with previous reports of social cognition deficits in PPA (Fittipaldi et al., 2019) and FTD-ALS (Savage et al., 2014) as well as bvFTD.

Importantly, we also found a decrease in emotion processing abilities in the late *C9orf72* mutation carriers (those within 5 years to symptom onset) when compared to controls, the other late presymptomatic carriers and the early *C9orf72* presymptomatic mutation carriers. This deficit was seen particularly on items of fear and sadness. This finding is consistent with other smaller studies showing subtle social cognitive deficits prior to symptom onset in genetic FTD (Jiskoot et al., 2016, 2018; Cheran et al., 2019). However, in prior studies, only presymptomatic *MAPT* and *GRN* mutation carriers have been studied, with deficits in social cognition only shown in *MAPT* but not *GRN* mutation carriers. The differences from our study (i.e. the lack of deficits shown in *MAPT* mutation carriers) may well be accounted for by a difference in the tests performed (in one study deficits were found in questionnaires rather than cognitive tests: Cheran et al., 2019), and the fact that in two of the studies, deficits were only detected longitudinally, and approaching phenoconversion (Jiskoot et al., 2016, 2018).

Impairment on tasks of social cognition is likely to involve breakdown of a number of processes within the brain. Consistent with this, previous studies of the neural correlates of social cognition deficits in sporadic FTD have shown an association of emotional processing difficulties with a variety of brain regions including frontal (particularly orbitofrontal), inferior temporal, and insula cortices as well as the amygdala (reviewed in Kumfor and Piguet, 2012; Couto et al., 2013). Similarly, theory of mind problems have also been associated with atrophy within a variety of areas in the brain including frontal cortex, temporal and insular regions (Adenzato et al., 2010; Agustus et al., 2015; Bertoux et al., 2014; Guevara et al., 2015). In our study, orbitofrontal cortex was fairly uniformly affected across each of the genetic groups – this region is known to be involved in complex social and emotional behaviour (Kringelbach, 2004; Rolls, 2004; Beer, John, Scabini, & Knight, 2006), particularly through a role in stimulus-reinforcement learning and processing of reward. The insula was similarly affected across the groups in both tasks – this region is a core hub of the salience network which is involved in a wide variety of social processes (Menon & Uddin, 2010; Uddin, Nomi, Hebert-Seropian, Ghaziri, & Boucher, 2017) such as interoception, the processing of emotional experiences and the awareness of positive and negative feelings (Craig, 2002), all required when trying to identify emotions and interpret social situations. Also previously reported is the association of the inferior and medial temporal lobe, particularly the amygdala, with social cognition deficits in FTD, areas known to be involved in the perception and recognition of facial emotions – this region was associated with performance on both the FER test (in *C9orf72* and *GRN* mutation carriers) and FP recognition tests (in *GRN* mutation carriers).

A novel finding in this study was the association of the basal ganglia, particularly the striatum (caudate, putamen and nucleus accumbens), with impairment of social cognition across all of the three genetic groups and tests, except for the *C9orf72* FP recognition test performance. This region has previously been associated with emotion recognition deficits, particularly negative emotions (Sprengelmeyer et al., 1997; Calder et al., 2004; Kemp et al., 2013), although in one study of emotion generation, the basal ganglia was associated with dysregulation of producing happy emotions (Sturm et al., 2015). Other studies of sporadic FTD have also shown an association of the basal ganglia with performance on implicit emotion processing tasks (Balconi et al., 2015), and empathy measures (Rankin et al., 2006; Shdo et al., 2017). Furthermore, neuroanatomically, the striatum is highly connected with frontal regions, with fronto-striatal circuits implicated in the early pathological processes in FTD (Yi et al., 2013; Sobue et al., 2018) and atrophy in the striatum found across all genetic subtypes of FTD (Bede et al., 2013; Rohrer et al., 2015; Cash et al., 2018). This work therefore provides support for the role of the basal ganglia in social cognitive abilities in genetic FTD.

A key strength of this study is the large sample size: whilst familial FTD is a relatively rare condition, by using data collected as part of GENFI, it allows investigation of a larger group of individuals with familial FTD including those in the presymptomatic period. Despite this, some groups remain with small sample sizes (particularly *MAPT* mutation carriers); the continuation of data collection as part of GENFI will help to overcome this problem. A further limitation of the study is the use of the mean age at onset within a family to estimate the number of years from likely symptom onset within an individual. As shown previously within the GENFI study (Moore et al., 2020), whilst there is a highly significant correlation between an individual's age at symptom onset and the mean age at symptom onset within the family in all three genetic mutations, the correlations are lower for *C9orf72* and *GRN* mutation carriers, making the estimate inexact. However, there are currently no better methods for estimating time from likely symptom onset at present, with future studies likely to benefit from the development of more precise measures of proximity to onset.

Given that structural neuroanatomical changes occur quite a number of years prior to symptom onset in each of the genetic groups (Rohrer et al., 2015) it may seem surprising that social cognitive deficits were only shown in one group (*C9orf72*) and in one test during the presymptomatic period. The question then arises as to whether the current tests are sensitive enough to detect the earliest social cognitive changes that occur, or whether social cognition deficits would still be found to occur only very late in the presymptomatic period or early in the symptomatic period even with other tasks. Further work is required to tease apart these two possibilities with the development and testing of novel social cognitive tasks within such presymptomatic cohorts both cross-sectionally and particularly longitudinally where one can identify individuals who phenoconvert. Such studies would enhance understanding of the timing and progression of social cognitive changes within genetic FTD.

In summary, this study demonstrates that the FER and FP recognition tests are able to identify deficits in emotion processing and theory of mind in familial cases of FTD across the three main genetic mutation groups, including during the late presymptomatic period in *C9orf72* mutation carriers. Furthermore, neuroanatomical regions known to be involved with social cognition were found to be correlated with performance on the tasks, with the novel finding of basal ganglia involvement in genetic FTD. This frontal-striatal-insula-temporal network is highly interconnected and forms part of a previously described social brain functional network (Adolphs, 2002; Pessoa, 2017) which allows people to interact with each other and learn social behaviours so that they can follow societal norms – factors lost in people in FTD. The FER and FP recognition tests may prove useful as cognitive markers in future clinical trials of FTD but further work is needed to understand the longitudinal change over time, with further refinement of tasks to more sensitively detect changes in the presymptomatic period.

## CRediT author statement

**Lucy L. Russell:** Conceptualization (supporting); Formal analysis (lead); Visualization (lead); Writing - Original Draft (lead); Writing - Review & Editing (equal). **Caroline V. Greaves:** Project administration (equal); Resources (supporting); Investigation (equal). **Martina Bocchetta:** Formal analysis (supporting); Writing - Review & Editing (equal). **Jennifer Nicholas:** Formal analysis (supporting); Writing - Review & Editing (equal). **Rhian S. Convery:** Data Curation (lead); Investigation (equal). **Katrina Moore:** Project administration (equal); Data Curation (Supporting); Investigation (equal); Resources (lead). **David M. Cash:** Formal analysis (supporting); Writing - Review & Editing (equal). **John van Swieten:** Resources (equal); Project administration (equal); Funding acquisition (equal); Investigation (supporting); Writing - Review & Editing (supporting). **Lize Jiskoot:** Resources (equal); Project administration (equal); Funding acquisition (equal); Investigation (supporting); Writing - Review & Editing (supporting). **Fermin Moreno:** Resources (equal); Project administration (equal); Funding acquisition (equal); Investigation (supporting); Writing - Review & Editing (supporting). **Raquel Sanchez-Valle:** Resources (equal); Project administration (equal); Funding acquisition (equal); Investigation (supporting); Writing - Review & Editing (supporting). **Barbara Borroni:** Resources (equal); Project administration (equal); Funding acquisition (equal); Investigation (supporting); Writing - Review & Editing (supporting). **Robert Laforce Jr:** Resources (equal); Project administration (equal); Funding acquisition (equal); Investigation (supporting); Writing - Review & Editing (supporting). **Mario Masellis:** Resources (equal); Project administration (equal); Funding acquisition (equal); Investigation (supporting); Writing - Review & Editing (supporting). **Maria Carmela Tartaglia:** Resources (equal); Project administration (equal); Funding acquisition (equal); Investigation (supporting); Writing - Review & Editing (supporting). **Caroline Graff:** Resources (equal); Project administration (equal); Funding acquisition (equal); Investigation (supporting); Writing - Review & Editing (supporting). **Emanuela Rotondo:** Resources (equal); Project administration (equal); Funding acquisition (equal); Investigation (supporting); Writing - Review & Editing (supporting). **Daniela Galimberti:** Resources (equal); Project administration (equal); Funding acquisition (equal); Investigation (supporting); Writing - Review & Editing (supporting). **James B Rowe:** Resources (equal); Project administration (equal); Funding acquisition (equal); Investigation (supporting); Writing - Review & Editing (supporting). **Elizabeth Finger:** Resources (equal); Project administration (equal); Funding acquisition (equal); Investigation (supporting); Writing - Review & Editing (supporting). **Matthis Synofzik:** Resources (equal); Project administration (equal); Funding acquisition (equal); Investigation (supporting); Writing - Review & Editing (supporting). **Rik Vandenberghe:** Resources (equal); Project administration (equal); Funding acquisition (equal); Investigation (supporting); Writing - Review & Editing (supporting). **Alexandre de Mendonça:** Resources (equal); Project administration (equal); Funding acquisition (equal); Investigation (supporting); Writing - Review & Editing (supporting). **Fabrizio Tagliavini:** Resources (equal); Project administration (equal); Funding acquisition (equal); Investigation (supporting); Writing - Review & Editing (supporting). **Isabel Santana:** Resources (equal); Project administration (equal); Funding acquisition (equal); Investigation (supporting); Writing - Review & Editing (supporting). **Simon Ducharme:** Resources (equal); Project administration (equal); Funding acquisition (equal); Investigation (supporting); Writing - Review & Editing (supporting). **Chris Butler:** Resources (equal); Project administration (equal); Funding acquisition (equal); Investigation (supporting); Writing - Review & Editing (supporting). **Alex Gerhard:** Resources (equal); Project administration (equal); Funding acquisition (equal); Investigation (supporting); Writing - Review & Editing (supporting). **Johannes Levin:** Resources (equal); Project administration (equal); Funding acquisition (equal); Investigation (supporting); Writing - Review & Editing (supporting). **Adrian Danek:** Resources (equal); Project administration (equal); Funding acquisition (equal); Investigation (supporting); Writing - Review & Editing (supporting). **Markus Otto:** Resources (equal); Project administration (equal); Funding acquisition (equal); Investigation (supporting); Writing - Review & Editing (supporting). **Jason D Warren:** Resources (equal); Project administration (equal); Funding acquisition (equal); Investigation (supporting); Writing - Review & Editing (supporting). **Jonathan D Rohrer:** Conceptualization (lead); Supervision (lead); Formal analysis (supporting); Writing - Review & Editing (equal); Project administration (equal); Funding acquisition (lead).

## Open practices

The study in this article earned an Open Data badge for transparent practices. Statistical analysis from this study will be made available on reasonable request.

## Appendix

**List of GENFI consortium authors:**

Martin N. Rossor ^ab^, Nick C. Fox ^ab^, Ione O.C. Woollacott ^ab^, Rachelle Shafei ^ab^, Carolin Heller ^ab,ac^, Rita Guerreiro ^ac^, Jose Bras ^ac^, David L. Thomas ^ad^, Simon Mead ^ae^, Lieke Meeter ^af^, Jessica Panman ^af^, Janne Papma ^af^, Jackie Poos ^af^, Rick van Minkelen ^ag^, Yolanda Pijnenburg ^ah^, Myriam Barandiaran ^ai,aj^, Begoña Indakoetxea ^ai,aj^, Alazne Gabilondo ^aj^, Mikel Tainta ^aj^, Maria de Arriba ^aj^, Ana Gorostidi ^aj^, Miren Zulaica ^aj^, Jorge Villanua ^ak^ Zigor Diaz^al^, Sergi Borrego-Ecija ^am^, Jaume Olives ^am^, Albert Lladó ^am^, Mircea Balasa ^am^, Anna Antonell ^am^, Nuria Bargallo ^an^, Enrico Premi ^ao^, Maura Cosseddu ^ao^, Stefano Gazzina ^ao^, Alessandro Padovani ^ao^, Roberto Gasparotti ^ap^, Silvana Archetti ^aq^, Sandra Black ^ar^, Sara Mitchell ^ar^, Ekaterina Rogaeva ^as^, Morris Freedman ^at^, Ron Keren ^au^, Daid Tang-Wai ^av^, Linn Öijerstedt ^aw^, Christin Andersson ^ax^, Vesna Jelic ^ay^, Hakan Thonberg ^az^, Andrea Arighi ^ba,bb^, Chiara Fenoglio ^ba,bb^, Elio Scarpini ^ba,bb^, Giorgio Fumagalli ^ba,bb,bc^, Thomas Cope ^bd^, Carolyn Timberlake ^bd^, Timothy Rittman ^bd^, Christen Shoesmith ^be^, Robart Bartha ^bf,bg^, Rosa Rademakers ^bh^, Carlo Wilke ^bi,bj^, Hans-Otto Karnarth ^bk^, Benjamin Bender ^bl^, Rose Bruffaerts ^bm^, Philip Vandamme ^bn^, Mathieu Vandenbulcke ^bo,bp^, Catarina B. Ferreira ^bq^, Gabriel Miltenberger ^br^, Carolina Maruta ^bs^, Ana Verdelho ^bt^, Sónia Afonso ^bu^, Ricardo Taipa ^bv^, Paola Caroppo ^bw^, Giuseppe Di Fede ^bw^, Giorgio Giaccone ^bw^, Cristina Muscio, ^bw^, Sara Prioni ^bw^, Veronica Redaelli ^bw^, Giacomina Rossi ^bw^, Pietro Tiraboschi ^bw^, Diana Duro^bx^, Maria R. Almeida ^bx^, Miguel Castelo-Branco ^bx^, Maria J. Leitão ^by^, Miguel Tabuas-Pereira ^bz^, Beatriz Santiago ^bz^, Serge Gauthier ^ca^, Pedro Rosa-Neto ^cb^, Michele Veldsman ^cc^, Paul Thompson ^cd^, Tobias Langheinrich ^cd^, Catharina Prix ^ce^, Tobias Hoegen ^ce^, Elisabeth Wlasich ^ce^, Sandra Loosli ^ce^, Sonja Schonecker ^ce^, Elisa Semler ^cf^, Sarah Anderl-Straub ^cf^.

**Affiliations:**

^ab^Dementia Research Centre, Department of Neurodegenerative Disease, UCL Institute of Neurology, Queen Square, London, UK.

^ac^Dementia Research Institute, Department of Neurodegenerative Disease, UCL Institute of Neurology, Queen Square, London, UK.

^ad^Neuroimaging Analysis Centre, Department of Brain Repair and Rehabilitation, UCL Institute of Neurology, Queen Square, London, UK.

^ae^MRC Prion Unit, Department of Neurodegenerative Disease, UCL Institute of Neurology, Queen Square, London, UK.

^af^Department of Neurology, Erasmus Medical Centre, Rotterdam, Netherlands.

^ag^Department of Clinical Genetics, Erasmus Medical Centre, Rotterdam, Netherlands.

^ah^Amsterdam University Medical Centre, Amsterdam VUmc, Amsterdam, Netherlands.

^ai^Cognitive Disorders Unit, Department of Neurology, Donostia University Hospital, San Sebastian, Gipuzkoa, Spain.

^aj^Neuroscience Area, Biodonostia Health Research Insitute, San Sebastian, Gipuzkoa, Spain.

^ak^OSATEK, University of Donostia, San Sebastian, Gipuzkoa, Spain.

^al^CITA Alzheimer, San Sebastian, Gipuzkoa, Spain.

^am^Alzheimer's Disease and Other Cognitive Disorders Unit, Neurology Service, Hospital Clínic, Barcelona, Spain.

^an^Imaging Diagnostic Center, Hospital Clínic, Barcelona, Spain.

^ao^Centre for Neurodegenerative Disorders, Neurology Unit, Department of Clinical and Experimental Sciences, University of Brescia, Brescia, Italy.

^ap^Neuroradiology Unit, University of Brescia, Brescia, Italy.

^aq^Biotechnology Laboratory, Department of Diagnostics, Spedali Civili Hospital, Brescia, Italy.

^ar^Sunnybrook Health Sciences Centre, Sunnybrook Research Institute, University of Toronto, Toronto, Canada.

^as^Tanz Centre for Research in Neurodegenerative Diseases, University of Toronto, Toronto, Canada.

^at^Baycrest Health Sciences, Rotman Research Institute, University of Toronto, Toronto, Canada.

^au^The University Health Network, Toronto Rehabilitation Institute, Toronto, Canada.

^av^The University Health Network, Krembil Research Institute, Toronto, Canada.

^aw^Department of Geriatric Medicine, Karolinska University Hospital-Huddinge, Stockholm, Sweden.

^ax^Department of Clinical Neuroscience, Karolinska Institutet, Stockholm, Sweden.

^ay^Division of Clinical Geriatrics, Karolinska Institutet, Stockholm, Sweden.

^az^Center for Alzheimer Research, Divison of Neurogeriatrics, Karolinska Institutet, Stockholm, Sweden.

^ba^Fondazione IRCCS Ca’ Granda Ospedale Maggiore Policlinico, Neurodegenerative Diseases Unit, Milan, Italy.

^bb^University of Milan, Centro Dino Ferrari, Milan, Italy.

^bc^Department of Neurosciences, Psychology, Drug Research and Child Health (NEUROFARBA), University of Florence, Florence, Italy.

^bd^Department of Clinical Neurosciences, University of Cambridge, Cambridge, UK.

^be^Department of Clinical Neurological Sciences, University of Western Ontario, London, Ontario Canada.

^bf^Department of Medical Biophysics, The University of Western Ontario, London, Ontario, Canada.

^bg^Centre for Functional and Metabolic Mapping, Robarts Research Institute, The University of Western Ontario, London, Ontario, Canada.

^bh^Department of Neuroscience, Mayo Clinic, Jacksonville, FL, USA.

^bi^Department of Neurodegenerative Diseases, Hertie-Institute for Clinical Brain Research and Center of Neurology, University of Tübingen, Tübingen, Germany.

^bj^Center for Neurodegenerative Diseases (DZNE), Tübingen, Germany.

^bk^Division of Neuropsychology, Hertie-Institute for Clinical Brain Research and Center of Neurology, University of Tübingen, Tübingen, Germany.

^bl^Department of Diagnostic and Interventional Neuroradiology, University of Tübingen, Tübingen, Germany.

^bm^Laboratory for Cognitive Neurology, Department of Neurosciences, KU Leuven, Leuven, Belgium.

^bn^Neurology Service, University Hospitals Leuven, Belgium, Laboratory for Neurobiology, VIB-KU Leuven Centre for Brain Research, Leuven, Belgium.

^bo^Geriatric Psychiatry Service, University Hospitals Leuven, Belgium.

^bp^Neuropsychiatry, Department of Neurosciences, KU Leuven, Leuven, Belgium.

^bq^Laboratory of Neurosciences, Institute of Molecular Medicine, Faculty of Medicine, University of Lisbon, Lisbon, Portugal.

^br^Faculty of Medicine, University of Lisbon, Lisbon, Portugal.

^bs^Laboratory of Language Research, Centro de Estudos Egas Moniz, Faculty of Medicine, University of Lisbon, Lisbon, Portugal.

^bt^Department of Neurosciences and Mental Health, Centro Hospitalar Lisboa Norte - Hospital de Santa Maria & Faculty of Medicine, University of Lisbon, Lisbon, Portugal.

^bu^Instituto Ciencias Nucleares Aplicadas a Saude, Universidade de Coimbra, Coimbra, Portugal.

^bv^Neuropathology Unit and Department of Neurology, Centro Hospitalar do Porto - Hospital de Santo António, Oporto, Portugal.

^bw^Fondazione IRCCS Istituto Neurologico Carlo Besta, Milano, Italy.

^bx^Faculty of Medicine, University of Coimbra, Coimbra, Portugal.

^by^Centre of Neurosciences and Cell Biology, Universidade de Coimbra, Coimbra, Portugal.

^bz^Neurology Department, Centro Hospitalar e Universitario de Coimbra, Coimbra, Portugal.

^ca^Alzheimer Disease Research Unit, McGill Centre for Studies in Aging, Department of Neurology & Neurosurgery, McGill University, Montreal, Québec, Canada.

^cb^Translational Neuroimaging Laboratory, McGill Centre for Studies in Aging, McGill University, Montreal, Québec, Canada.

^cc^Nuffield Department of Clinical Neurosciences, Medical Sciences Division, University of Oxford, Oxford, UK.

^cd^Faculty of Biology, Medicine and Health, Division of Neuroscience and Experimental Psychology, University of Manchester, Manchester, UK.

^ce^Neurologische Klinik, Ludwig-Maximilians-Universität München, Munich, Germany.

^cf^Department of Neurology, University of Ulm, Ulm, Germany.
