# Supplementary material for: Social cognition impairment in genetic frontotemporal dementia within the GENFI cohort
Source: Cortex. 2020 Dec;133:384–98. doi: 10.1016/j.cortex.2020.08.023 (PMC7754789; doi:10.1016/j.cortex.2020.08.023)
Supplement: Multimedia component 1 [file mmc1.docx]

# Supplementary data

Table S1: Percentage of participants that scored 0, 0.5, 1, 2, or 3 on the FTLD-CDR-Global, behaviour and language subscores. The first percentage denotes the scores in relation to those with a FER score and those in brackets relates to those with a score on the FP task.

|  | | **Controls** | **C9orf72** | | | **GRN** | | | **MAPT** | | |
| --- | --- | --- | --- | --- | --- | --- | --- | --- | --- | --- | --- |
|  |  |  | **Early PS** | **Late PS** | **Symptomatic** | **Early PS** | **Late PS** | **Symptomatic** | **Early PS** | **Late PS** | **Symptomatic** |
| N | | 264 (245) | 81 | 25 (24) | 53 (45) | 93 | 29 (30) | 32 (22) | 37 (30) | 12 | 18 (12) |
| FTLD-CDR-Global | 0 | 82% (82%) | 77% | 76% (75%) | 4% (3%) | 88% | 79% (80%) |  | 81% (81%) | 83% |  |
|  | 0.5 | 16% (16%) | 21% | 20% (21%) | 11% (11%) | 12% | 21% (20%) | 6% (5%) | 14% (14%) | 17% | 17% (25%) |
|  | 1 | 2% (2%) | 2% | 4% (4%) | 19% (20%) |  |  | 38% (41%) | 5% (5%) |  | 11% (8%) |
|  | 2 |  |  |  | 40% (42%) |  |  | 31% (32%) |  |  | 50% (42%) |
|  | 3 |  |  |  | 26% (24%) |  |  | 25% (23%) |  |  | 22% (25%) |
| Behaviour  score | 0 | 96% (96%) | 90% | 92% (92%) | 13% (13%) | 96% | 90% (90%) | 19% (14%) | 81% (94%) | 92% | 39% (16%) |
|  | 0.5 | 3% (3%) | 10% |  | 9% (9%) | 4% | 7% (10%) | 9% (4%) | 11% (3%) | 8% | 17% (17%) |
|  | 1 | 1% (1%) |  | 8% (8%) | 25% (24%) |  | 3% (0%) | 25% (50%) | 8% (3%) |  | 33% (17%) |
|  | 2 |  |  |  | 43% (45%) |  |  | 34% (32%) |  |  | 11% (50%) |
|  | 3 |  |  |  | 10% (9%) |  |  | 13% (0%) |  |  |  |
| Language  score | 0 | 90% (91%) | 90% | 92% (92%) | 28% (29%) | 96% | 90% (90%) | 19% (23%) | 81% (81%) | 92% | 39% (33%) |
|  | 0.5 | 8% (7%) | 10% | 4% (4%) | 19% (20%) | 4% | 7% (7%) | 9% (13%) | 11% (11%) |  | 17% (25%) |
|  | 1 | 2% (2%) |  | 4% (4%) | 36% (36%) |  | 3% (3%) | 25% (32%) | 8% (8%) | 8% | 33% (25%) |
|  | 2 |  |  |  | 15% (13%) |  |  | 34% (23%) |  |  | 11% (17%) |
|  | 3 |  |  |  | 2% (2%) |  |  | 13% (9%) |  |  |  |

Table S2 - Facial Emotion Recognition test scores in controls by age.

| **Age group (years)** | **All** | | | **Females** | | | **Males** | | |
| --- | --- | --- | --- | --- | --- | --- | --- | --- | --- |
|  | **Number of participants** | **Mean** | **Standard deviation** | **Number of participants** | **Mean** | **Standard deviation** | **Number of participants** | **Mean** | **Standard deviation** |
| 18.1-29.9 | 29 | 28.7 | 2.9 | 15 | 28.4 | 3.1 | 14 | 29.0 | 2.6 |
| 30.0-39.9 | 59 | 28.8 | 3.2 | 34 | 28.9 | 3.0 | 25 | 28.6 | 3.6 |
| 40.0-49.9 | 67 | 29.6 | 2.9 | 37 | 29.9 | 2.7 | 30 | 29.2 | 3.1 |
| 50.0-59.9 | 46 | 28.5 | 3.3 | 31 | 29.3 | 3.4 | 15 | 26.8 | 2.4 |
| 60.0-69.9 | 39 | 27.6 | 3.5 | 25 | 28.4 | 3.3 | 14 | 26.3 | 3.6 |
| 70.0-85.0 | 6 | 27.8 | 2.5 | 1 | 28.0 | - | 5 | 27.8 | 2.8 |

Table S3 – Facial Emotion Recognition test scores in controls by education.

| **Education group** | **All** | | | **Females** | | | **Males** | | |
| --- | --- | --- | --- | --- | --- | --- | --- | --- | --- |
|  | **Number of participants** | **Mean** | **Standard deviation** | **Number of participants** | **Mean** | **Standard deviation** | **Number of participants** | **Mean** | **Standard deviation** |
| 0-9 | 22 | 28.0 | 3.1 | 9 | 27.6 | 3.6 | 13 | 28.4 | 2.8 |
| 10-12 | 47 | 28.0 | 4.0 | 23 | 28.8 | 3.2 | 24 | 27.2 | 4.6 |
| 13-16 | 122 | 28.9 | 3.0 | 78 | 29.2 | 3.1 | 44 | 28.4 | 2.7 |
| >17 | 55 | 29.2 | 2.7 | 33 | 29.5 | 2.7 | 22 | 28.9 | 2.7 |

Table S4 – Facial Emotion Recognition test score in controls – cumulative frequency.

| **FER test score** | **Number of participants** | **Cumulative frequency (%)** |
| --- | --- | --- |
| 19 | 3 | 1.2 |
| 20 | 3 | 2.4 |
| 21 | 1 | 2.8 |
| 22 | 2 | 3.7 |
| 23 | 7 | 6.5 |
| 24 | 9 | 10.2 |
| 25 | 15 | 16.3 |
| 26 | 16 | 22.8 |
| 27 | 16 | 29.3 |
| 28 | 29 | 41.1 |
| 29 | 36 | 55.7 |
| 30 | 33 | 69.1 |
| 31 | 28 | 80.5 |
| 32 | 24 | 90.2 |
| 33 | 16 | 96.7 |
| 34 | 8 | 100.0 |

Table S5 – Adjusted mean differences in FER test score between the groups with p-values (significant in bold) and 95% bias-corrected confidence intervals.

|  |  | **Control** | | ***C9orf72*** | | | | | | ***GRN*** | | | | | | ***MAPT*** | | | | | |
| --- | --- | --- | --- | --- | --- | --- | --- | --- | --- | --- | --- | --- | --- | --- | --- | --- | --- | --- | --- | --- | --- |
|  |  |  |  | **Early PS** | | **Late PS** | | **Symptomatic** | | **Early PS** | | **Late PS** | | **Symptomatic** | | **Early PS** | | **Late PS** | | **Symptomatic** | |
| **Control** |  |  | | -0.02 | | **-1.83** | | **-9.03** | | 0.23 | | 0.21 | | **-7.47** | | 0.29 | | 0.95 | | **-5.95** | |
|  |  |  |  | -0.80 | 0.76 | **-2.93** | **-0.73** | **-11.06** | **-7.00** | -0.64 | 1.11 | -1.81 | 2.24 | **-9.83** | **-5.10** | -0.92 | 1.50 | -0.30 | 2.19 | **-9.25** | **-2.66** |
| **C9ORF72** | **Early PS** |  | |  | | **-1.81** | | **-9.01** | | 0.25 | | 0.23 | | **-7.45** | | 0.31 | | 0.97 | | **-5.93** | |
|  |  |  |  |  |  | **-3.08** | **-0.54** | **-11.17** | **-6.85** | -0.87 | 1.37 | -1.95 | 2.42 | **-9.95** | **-4.95** | -0.99 | 1.61 | -0.47 | 2.40 | **-9.29** | **-2.58** |
|  | **Late PS** |  | |  | |  | | **-7.20** | | **2.06** | | 2.04 | | **-5.64** | | **2.12** | | **2.77** | | **-4.12** | |
|  |  |  |  |  |  |  |  | **-9.34** | **-5.07** | **0.71** | **3.41** | -0.28 | 4.36 | **-8.15** | **-3.13** | **0.54** | **3.71** | **1.24** | **4.31** | **-7.53** | **-0.72** |
|  | **Symptomatic** |  | |  | |  | |  | | **9.26** | | **9.24** | | 1.56 | | **9.32** | | **9.98** | | 3.08 | |
|  |  |  |  |  |  |  |  |  |  | **7.05** | **11.47** | **6.46** | **12.02** | -1.41 | 4.53 | **6.90** | **11.75** | **7.63** | **12.32** | -0.67 | 6.82 |
| **GRN** | **Early PS** |  | |  | |  | |  | |  | | -0.02 | | **-7.70** | | 0.06 | | 0.71 | | **-6.18** | |
|  |  |  |  |  |  |  |  |  |  |  |  | -1.99 | 1.95 | **-10.09** | **-5.31** | -1.32 | 1.45 | -0.79 | 2.22 | **-9.58** | **-2.79** |
|  | **Late PS** |  | |  | |  | |  | |  | |  | | **-7.68** | | 0.08 | | 0.73 | | **-6.17** | |
|  |  |  |  |  |  |  |  |  |  |  |  |  |  | **-10.67** | **-4.70** | -2.26 | 2.42 | -1.66 | 3.13 | **-9.94** | **-2.39** |
|  | **Symptomatic** |  | |  | |  | |  | |  | |  | |  | | **7.76** | | **8.42** | | 1.52 | |
|  |  |  |  |  |  |  |  |  |  |  |  |  |  |  |  | **5.15** | **10.37** | **5.82** | **11.01** | -2.48 | 5.52 |
| **MAPT** | **Early PS** |  | |  | |  | |  | |  | |  | |  | |  | | 0.65 | | **-6.25** | |
|  |  |  |  |  |  |  |  |  |  |  |  |  |  |  |  |  |  | -0.91 | 2.21 | **-9.78** | **-2.71** |
|  | **Late PS** |  | |  | |  | |  | |  | |  | |  | |  | |  | | **-6.90** | |
|  |  |  |  |  |  |  |  |  |  |  |  |  |  |  |  |  |  |  |  | **-10.55** | **-3.25** |
|  | **Symptomatic** |  | |  | |  | |  | |  | |  | |  | |  | |  | |  | |
|  |  |  |  |  |  |  |  |  |  |  |  |  |  |  |  |  |  |  |  |  |  |

Table S6 – Analysis of performance on the Facial Emotion Recognition test by phenotype within the symptomatic mutation carriers. *Note that other phenotypes were not included due to low sample sizes (AD: N = 1; Dementia-NOS: N = 2; Other: N = 2; PSP: N = 1).*

| **Diagnosis** | **N** | **Genetic groups** | **FER test score**  ***Mean (SD)*** |
| --- | --- | --- | --- |
| Controls | 210 |  | 28.5 (3.3) |
| bvFTD | 62 | C9orf72 = 32  GRN = 15  MAPT = 15 | 19.6 (6.3) |
| PPA | 15 | C9orf72 = 1  GRN = 14  MAPT = 0 | 22.0 (6.4) |
| FTD-ALS/ALS | 8 | *C9orf72* = 8  GRN = 0  MAPT = 0 | 18.4 (8.1) |

Table S7 – Adjusted mean differences in FER test score between the phenotypic groups with p-values (significant in bold) and 95% bias-corrected confidence intervals.

| **Group comparisons** | | | **Adjusted mean difference** | **p-values** | **95% confidence intervals** | |
| --- | --- | --- | --- | --- | --- | --- |
| Control | vs | bvFTD | **7.6** | **< 0.001** | **5.9** | **9.3** |
| Control | vs | PPA | **5.7** | **< 0.001** | **2.7** | **8.8** |
| Control | vs | FTD-ALS/ALS | **8.5** | **0.004** | **2.7** | **14.2** |
| bvFTD | vs | PPA | -1.9 | 0.247 | -5.1 | 1.3 |
| bvFTD | vs | FTD-ALS/ALS | 0.9 | 0.768 | -5.0 | 6.7 |
| PPA | vs | FTD-ALS/ALS | 2.8 | 0.393 | -3.6 | 9.1 |

Table S8 – Positive neuroanatomical correlates of grey matter volume on the FER test in each genetic group – italics indicate uncorrected results at p<0.001.

| **Genetic group** | **Region** | **Cluster** | **T** | **Peak** | | **Co-ordinates (mm)** | | |
| --- | --- | --- | --- | --- | --- | --- | --- | --- |
|  |  |  |  | p(FWE-corr) | p(unc.) | x | y | z |
| ***C9orf72*** | Left putamen | 323 | 5.63 | 0.002 | < 0.001 | -27 | 4 | 6 |
|  | Left middle frontal gyrus | 70 | 5.54 | 0.003 | < 0.001 | -24 | 50 | 4 |
|  | Right anterior insula | 31 | 5.53 | 0.004 | < 0.001 | 44 | -4 | 4 |
|  | Left posterior insula | 41 | 5.52 | 0.004 | < 0.001 | -42 | -14 | -3 |
|  | Left orbitofrontal | 26 | 5.28 | 0.01 | < 0.001 | -12 | 45 | -18 |
|  | Right amygdala | 19 | 5.21 | 0.013 | < 0.001 | 18 | -8 | -12 |
|  | Left caudate | 125 | 5.12 | 0.018 | < 0.001 | -12 | 18 | 9 |
|  | Left basal ganglia, insula, orbitofrontal cortex, hippocampus and amygdala | 8185 | 7.69 | < 0.001 | < 0.001 | -22 | 4 | -4 |
|  | Left inferior temporal gyrus | 238 | 6.59 | < 0.001 | < 0.001 | -51 | -38 | -18 |
|  | Right superior frontal gyrus | 73 | 6.38 | < 0.001 | < 0.001 | 21 | 14 | 54 |
|  | Right middle frontal gyrus | 250 | 6.26 | < 0.001 | < 0.001 | 34 | 44 | 24 |
|  | Left inferior frontal gyrus | 724 | 6.23 | < 0.001 | < 0.001 | -46 | 40 | 2 |
|  | Left superior frontal gyrus | 234 | 6.13 | < 0.001 | < 0.001 | -3 | 32 | 50 |
|  | Left middle temporal gyrus | 77 | 6.02 | < 0.001 | < 0.001 | -64 | -28 | -3 |
|  | Right caudate | 371 | 5.73 | 0.002 | < 0.001 | 12 | 8 | 16 |
|  | Left temporal pole | 211 | 5.61 | 0.003 | < 0.001 | -32 | 6 | -33 |
|  | Right middle frontal gyrus | 127 | 5.52 | 0.004 | < 0.001 | 32 | 51 | -2 |
| ***GRN*** | Left insula | 30 | 5.42 | 0.006 | < 0.001 | -46 | -18 | 20 |
|  | Right superior frontal gyrus | 35 | 5.39 | 0.007 | < 0.001 | 10 | 44 | -6 |
|  | Right insula | 55 | 5.35 | 0.008 | < 0.001 | 42 | 26 | 4 |
|  | Left thalamus | 19 | 5.32 | 0.009 | < 0.001 | -8 | -26 | 14 |
|  | Right superior frontal gyrus | 44 | 5.31 | 0.009 | < 0.001 | 9 | 56 | 8 |
|  | Right superior frontal gyrus | 15 | 5.23 | 0.012 | < 0.001 | 18 | 48 | 32 |
|  | Left middle frontal gyrus | 28 | 5.18 | 0.015 | < 0.001 | -48 | 22 | 26 |
|  | Right orbitofrontal cortex | 30 | 5.17 | 0.016 | < 0.001 | 45 | 50 | -9 |
|  | Left middle frontal gyrus | 17 | 5.16 | 0.017 | < 0.001 | -39 | 15 | 52 |
|  | Left supplementary motor cortex | 20 | 5.16 | 0.017 | < 0.001 | -6 | 10 | 54 |
|  | Right middle cingulate gyrus | 34 | 5.15 | 0.017 | < 0.001 | 2 | -8 | 40 |
|  | Left insula | 106 | 5.12 | 0.019 | < 0.001 | -42 | -6 | 14 |
|  | Right orbitofrontal cortex | 39 | 5.1 | 0.02 | < 0.001 | 34 | 39 | -10 |
|  | Left middle frontal gyrus | 17 | 5 | 0.03 | < 0.001 | -38 | 8 | 27 |
|  | Left basal ganglia | 18 | 5.58 | 0.023 | < 0.001 | -14 | 12 | -9 |
|  | Left orbitofrontal cortex | *6* | *5.46* | *0.033* | *< 0.001* | *-9* | *24* | *-22* |
|  | *Right orbitofrontal cortex* | *199* | *4.93* | *0.149* | *< 0.001* | *26* | *21* | *-14* |
|  | *Right middle temporal gyrus* | *291* | *4.7* | *0.266* | *< 0.001* | *52* | *-12* | *-22* |
|  | *Left middle temporal gyrus* | *142* | *4.59* | *0.34* | *< 0.001* | *-54* | *-27* | *-14* |
| ***MAPT*** | *Right putamen* | *513* | *4.58* | *0.351* | *< 0.001* | *16* | *20* | *-12* |
|  | *Right superior frontal gyrus* | *216* | *4.47* | *0.44* | *< 0.001* | *16* | *12* | *63* |
|  | *Left superior frontal gyrus* | *69* | *4.45* | *0.457* | *< 0.001* | *-22* | *26* | *51* |
|  | *Left insula* | *122* | *4.06* | *0.818* | *< 0.001* | *-33* | *0* | *15* |
|  | *Left fusiform gyrus* | *100* | *4.03* | *0.841* | *< 0.001* | *-40* | *-52* | *-10* |
|  | *Left inferior temporal gyrus* | *78* | *3.89* | *0.926* | *< 0.001* | *-50* | *-24* | *-22* |
|  | *Left insula* | *103* | *3.81* | *0.957* | *< 0.001* | *-27* | *18* | *4* |

Table S9 - Faux Pas recognition test scores in controls by language – Swedish control group scored significantly higher than all other languages apart from Italian; the Italian control group scored significantly higher than all groups apart from Swedish and English groups; the English control group scored significantly higher than the Spanish group (all p<0.05).

|  | **FP recognition test language version** | **Number of participants** | **Mean** | **SD** |  |
| --- | --- | --- | --- | --- | --- |
|  | **English** | 59 | 36.1 | 4.4 |  |
|  | **Italian** | 37 | 36.6 | 3.6 |  |
|  | **Dutch** | 60 | 34.2 | 4.6 |  |
|  | **Swedish** | 11 | 38.2 | 1.9 |  |
|  | **French** | 29 | 34.9 | 4.1 |  |
|  | **Spanish** | 29 | 33.2 | 6.0 |  |
|  | **German** | 12 | 32.3 | 3.9 |  |
|  | **Portuguese** | 8 | 33.8 | 7.0 |  |

Table S10 – Faux Pas recognition test score in controls by age.

| **Age group (years)** | **All** | | | **Females** | | | **Males** | | |
| --- | --- | --- | --- | --- | --- | --- | --- | --- | --- |
|  | **Number of participants** | **Mean** | **Standard deviation** | **Number of participants** | **Mean** | **Standard deviation** | **Number of participants** | **Mean** | **Standard deviation** |
| 18.1-29.9 | 29 | 37.3 | 3.0 | 15 | 37.1 | 3.4 | 14 | 37.5 | 2.7 |
| 30.0-39.9 | 59 | 35.7 | 4.6 | 34 | 35.7 | 4.7 | 25 | 35.8 | 4.5 |
| 40.0-49.9 | 67 | 34.9 | 4.4 | 37 | 36.0 | 4.3 | 30 | 33.5 | 4.2 |
| 50.0-59.9 | 45 | 34.9 | 5.1 | 30 | 35.5 | 5.0 | 15 | 33.5 | 5.3 |
| 60.0-69.9 | 39 | 33.7 | 5.1 | 25 | 34.6 | 5.4 | 14 | 32.0 | 3.9 |
| 70.0-85.0 | 6 | 31.5 | 7.0 | 1 | 33.0 | . | 5 | 31.2 | 7.8 |

Table S11 – Faux Pas recognition test scores in controls by education.

| **Education group** | **All** | | | **Females** | | | **Males** | | |
| --- | --- | --- | --- | --- | --- | --- | --- | --- | --- |
|  | **Number of participants** | **Mean** | **Standard deviation** | **Number of participants** | **Mean** | **Standard deviation** | **Number of participants** | **Mean** | **Standard deviation** |
| 0-9 | 22 | 32.8 | 6.2 | 9 | 32.9 | 7.3 | 13 | 32.8 | 5.7 |
| 10-12 | 47 | 34.4 | 4.6 | 23 | 35.4 | 5.1 | 24 | 33.4 | 3.9 |
| 13-16 | 121 | 35.5 | 4.3 | 77 | 36.1 | 3.6 | 44 | 34.4 | 5.2 |
| >17 | 55 | 35.9 | 4.8 | 33 | 35.8 | 5.5 | 22 | 35.9 | 3.7 |

Table S12 – Faux Pas recognition test score in controls – cumulative frequency.

| **FP recognition test score** | **Number of participants** | **Cumulative frequency (%)** |
| --- | --- | --- |
| 19 | 2 | 0.8 |
| 20 | 0 | 0.8 |
| 21 | 1 | 1.2 |
| 22 | 3 | 2.4 |
| 23 | 1 | 2.9 |
| 24 | 1 | 3.3 |
| 25 | 3 | 4.5 |
| 26 | 4 | 6.1 |
| 27 | 4 | 7.8 |
| 28 | 5 | 9.8 |
| 29 | 5 | 11.8 |
| 30 | 9 | 15.5 |
| 31 | 13 | 20.8 |
| 32 | 12 | 25.7 |
| 33 | 18 | 33.1 |
| 34 | 21 | 41.6 |
| 35 | 13 | 46.9 |
| 36 | 9 | 50.6 |
| 37 | 20 | 58.8 |
| 38 | 21 | 67.3 |
| 39 | 26 | 78.0 |
| 40 | 54 | 100.0 |

Table S13 – Adjusted mean differences in FP recognition test score between the groups with p-values (significant in bold) and 95% bias-corrected confidence intervals.

|  |  | **Control** | | ***C9orf72*** | | | | | | ***GRN*** | | | | | | ***MAPT*** | | | | | |
| --- | --- | --- | --- | --- | --- | --- | --- | --- | --- | --- | --- | --- | --- | --- | --- | --- | --- | --- | --- | --- | --- |
|  |  |  |  | **Early PS** | | **Late PS** | | **Symptomatic** | | **Early PS** | | **Late PS** | | **Symptomatic** | | **Early PS** | | **Late PS** | | **Symptomatic** | |
| **Control** |  |  | | -0.77 | | -1.89 | | **-10.76** | | 0.54 | | 1.60 | | **-13.83** | | -0.61 | | -0.10 | | **-5.56** | |
|  |  |  |  | -2.11 | 0.58 | -4.95 | 1.17 | **-13.77** | **-7.76** | -0.60 | 1.67 | -0.03 | 3.24 | **-18.77** | **-8.89** | -2.20 | 0.97 | -2.78 | 2.57 | **-9.21** | **-1.92** |
| **C9ORF72** | **Early PS** |  | |  | | -1.13 | | **-10.00** | | 1.30 | | **2.37** | | **-13.07** | | 0.15 | | 0.66 | | **-4.80** | |
|  |  |  |  |  |  | -4.35 | 2.10 | **-13.15** | **-6.85** | -0.27 | 2.88 | **0.35** | **4.39** | **-18.21** | **-7.92** | -1.61 | 1.91 | -2.22 | 3.54 | **-8.70** | **-0.89** |
|  | **LatePS** |  | |  | |  | | **-8.87** | | 2.43 | | **3.50** | | **-11.94** | | 1.28 | | 1.79 | | -3.67 | |
|  |  |  |  |  |  |  |  | **-12.95** | **-4.80** | -0.79 | 5.65 | **0.26** | **6.73** | **-17.85** | **-6.03** | -2.10 | 4.65 | -2.13 | 5.71 | -8.27 | 0.93 |
|  | **Symptomatic** |  | |  | |  | |  | | **11.30** | | **12.37** | | -3.07 | | **10.15** | | **10.66** | | **5.20** | |
|  |  |  |  |  |  |  |  |  |  | **8.11** | **14.49** | **9.13** | **15.60** | -8.81 | 2.67 | **6.79** | **13.51** | **6.82** | **14.51** | **0.59** | **9.81** |
| **GRN** | **Early PS** |  | |  | |  | |  | |  | | 1.07 | | **-14.37** | | -1.15 | | -0.64 | | **-6.10** | |
|  |  |  |  |  |  |  |  |  |  |  |  | -0.54 | 2.67 | **-19.18** | **-9.56** | -2.84 | 0.54 | -3.51 | 2.23 | **-9.97** | **-2.23** |
|  | **LatePS** |  | |  | |  | |  | |  | |  | | **-15.44** | | **-2.22** | | -1.71 | | **-7.17** | |
|  |  |  |  |  |  |  |  |  |  |  |  |  |  | **-20.24** | **-10.63** | **-4.33** | **-0.11** | -4.61 | 1.19 | **-10.99** | **-3.34** |
|  | **Symptomatic** |  | |  | |  | |  | |  | |  | |  | | **13.22** | | **13.73** | | **8.27** | |
|  |  |  |  |  |  |  |  |  |  |  |  |  |  |  |  | **8.10** | **18.34** | **8.10** | **19.36** | **2.01** | **14.53** |
| **MAPT** | **Early PS** |  | |  | |  | |  | |  | |  | |  | |  | | 0.51 | | -4.95 | |
|  |  |  |  |  |  |  |  |  |  |  |  |  |  |  |  |  |  | -2.31 | 3.33 | -8.87 | -1.02 |
|  | **Late PS** |  | |  | |  | |  | |  | |  | |  | |  | |  | | **-5.46** | |
|  |  |  |  |  |  |  |  |  |  |  |  |  |  |  |  |  |  |  |  | **-9.00** | **-1.92** |
|  | **Symptomatic** |  | |  | |  | |  | |  | |  | |  | |  | |  | |  | |
|  |  |  |  |  |  |  |  |  |  |  |  |  |  |  |  |  |  |  |  |  |  |

Table S14 – Analysis of performance on the Faux Pas recognition test by phenotype within the symptomatic mutation carriers. *Note that other phenotypes were not included due to low sample sizes (AD: N = 1; Dementia-NOS: N = 1; Other: N = 2; PSP: N = 1).*

| **Diagnosis** | **N** | **Genetic groups** | **FP recognition test score**  ***Mean (SD)*** |
| --- | --- | --- | --- |
| Controls | 209 |  | 35.1 (4.6) |
| bvFTD | 50 | *C9orf72* = 27  *GRN* = 12  *MAPT* = 11 | 23.1 (10.0) |
| PPA | 8 | *C9orf72* = 1  *GRN* = 7  *MAPT* = 0 | 21.8 (14.6) |
| FTD-ALS/ALS | 6 | *C9orf72* = 6  *GRN* = 0  *MAPT* = 0 | 21.1 (12.1) |

Table S15 – Adjusted mean differences in FP recognition test score between the phenotypic groups with p-values (significant in bold) and 95% bias-corrected confidence intervals.

| **Group comparisons** | | | **Adjusted mean difference** | **p-values** | **95% confidence intervals** | |
| --- | --- | --- | --- | --- | --- | --- |
| Control | vs | bvFTD | **9.9** | **<0.001** | **7.1** | **12.7** |
| Control | vs | PPA | **11.4** | **0.008** | **3.0** | **19.8** |
| Control | vs | FTD-ALS/ALS | **9.7** | **0.044** | **0.2** | **19.2** |
| bvFTD | vs | PPA | 1.5 | 0.735 | -7.3 | 10.4 |
| bvFTD | vs | FTD-ALS/ALS | -0.2 | 0.974 | -9.7 | 9.4 |
| PPA | vs | FTD-ALS/ALS | -1.7 | 0.789 | -14.0 | 10.6 |

Table S16 – Positive neuroanatomical correlates of grey matter volume on the FP recognition test in each genetic group – italics indicate uncorrected results at p<0.001.

| **Genetic group** | **Region** | **Cluster** | **T** | **Peak** | | **Co-ordinates (mm)** | | |
| --- | --- | --- | --- | --- | --- | --- | --- | --- |
|  |  |  |  | p(FWE-corr) | p(unc) | x | y | z |
| ***C9orf72*** | Left precuneus | 274 | 6.11 | < 0.001 | < 0.001 | -3 | -68 | 24 |
|  | Left middle temporal gyrus | 59 | 5.58 | 0.002 | < 0.001 | -57 | -56 | 12 |
|  | Left middle occipital gyrus | 30 | 5.4 | 0.005 | < 0.001 | -33 | -93 | 8 |
|  | Right insula | 44 | 5.32 | 0.006 | < 0.001 | 45 | -2 | 2 |
|  | Left occipital lobe | 38 | 5.27 | 0.008 | < 0.001 | -18 | -99 | 3 |
|  | Left superior frontal gyrus | 25 | 5.24 | 0.009 | < 0.001 | -4 | 63 | 0 |
|  | Right superior occipital gyrus | 32 | 5.15 | 0.012 | < 0.001 | 28 | -90 | 16 |
|  | Left lingual gyrus | 37 | 5.1 | 0.015 | < 0.001 | -24 | -58 | -8 |
|  | Right planum polare | 35 | 5.04 | 0.019 | < 0.001 | 40 | -3 | -18 |
|  | Right fusiform gyrus | 64 | 5.04 | 0.019 | < 0.001 | 36 | -32 | -21 |
|  | Left: basal ganglia, orbitofrontal cortex, amygdala and hippocampus | 7509 | 8.03 | < 0.001 | < 0.001 | -10 | 9 | 9 |
|  | Left superior frontal gyrus | 891 | 6.68 | < 0.001 | < 0.001 | -6 | 51 | 18 |
|  | Left middle temporal gyrus | 131 | 6.6 | < 0.001 | < 0.001 | -64 | -26 | -4 |
|  | Right caudate | 621 | 6.53 | < 0.001 | < 0.001 | 12 | 6 | 16 |
|  | Left middle frontal gyrus | 429 | 6.33 | < 0.001 | < 0.001 | -34 | 32 | 42 |
|  | Left superior frontal gyrus | 155 | 6.03 | < 0.001 | < 0.001 | -4 | 42 | 36 |
|  | Right middle frontal gyrus | 153 | 6.02 | 0.001 | < 0.001 | 36 | 45 | 24 |
|  | Right middle frontal gyrus | 138 | 5.85 | 0.001 | < 0.001 | 34 | 57 | 4 |
| ***GRN*** | Left superior frontal gyrus | 57 | 5.8 | 0.001 | < 0.001 | -24 | 58 | 6 |
|  | Right orbitofrontal cortex | 148 | 5.72 | 0.002 | < 0.001 | 21 | 28 | -18 |
|  | Left supramarginal gyrus | 41 | 5.67 | 0.002 | < 0.001 | -58 | -40 | 30 |
|  | Left middle frontal gyrus | 57 | 5.58 | 0.003 | < 0.001 | -38 | 15 | 52 |
|  | Left precentral gyrus | 94 | 5.55 | 0.004 | < 0.001 | -44 | 6 | 28 |
|  | Left supramarginal gyrus | 16 | 5.47 | 0.005 | < 0.001 | -48 | -51 | 42 |
|  | Left precuneus | 79 | 5.47 | 0.005 | < 0.001 | -2 | -60 | 28 |
|  | Left middle frontal gyrus | 46 | 5.46 | 0.005 | < 0.001 | -33 | -3 | 58 |
|  | Right hippocampus | 22 | 5.43 | 0.006 | < 0.001 | 33 | -14 | -12 |
|  | Left temporal pole | 34 | 5.39 | 0.007 | < 0.001 | -39 | 18 | -27 |
|  | Right precuneus | 19 | 5.3 | 0.01 | < 0.001 | 2 | -45 | 40 |
|  | Left posterior insula | 22 | 5.27 | 0.011 | < 0.001 | -40 | -15 | -2 |
|  | Left inferior frontal gyrus | 43 | 5.26 | 0.012 | < 0.001 | -44 | 42 | 3 |
|  | Left fusiform gyrus | 40 | 5.23 | 0.013 | < 0.001 | -26 | -9 | -40 |
|  | Left precuneus | 32 | 5.14 | 0.019 | < 0.001 | -9 | -52 | 38 |
|  | Right superior frontal gyrus | 17 | 5.1 | 0.022 | < 0.001 | 8 | 42 | 21 |
|  | Left middle frontal gyrus | 18 | 5.05 | 0.027 | < 0.001 | -30 | 10 | 56 |
| ***MAPT*** | *Left basal ganglia and left orbitofrontal cortex* | *902* | *4.85* | *0.209* | *< 0.001* | *-10* | *21* | *-22* |
|  | *Right fusiform gyrus* | *130* | *3.65* | *0.993* | *< 0.001* | *8* | *20* | *-18* |
